# The Role of Principal and Secondary Diagnoses of Hospitalized Eye Trauma: A Nationwide Cohort in Taiwan, 1996-2010

**DOI:** 10.1371/journal.pone.0123348

**Published:** 2015-04-17

**Authors:** Jiahn-Shing Lee, Meng-Jiun Chiou, Feng-Ling Teng, Lai-Chu See

**Affiliations:** 1 Department of Ophthalmology, Chang Gung Memorial Hospital & Chang Gung University, Taoyuan City, Taiwan; 2 Department of Public Health, College of Medicine, Chang Gung University, Taoyuan City, Taiwan; 3 Department of Nursing, Chang Gung University of Science and Technology, Taoyuan City, Taiwan; 4 Biostatistics Core Laboratory, Molecular Medicine Research Center, Chang Gung University, Taoyuan City, Taiwan; National Taiwan University Hospital, TAIWAN

## Abstract

**Purpose:**

To estimate the rate of hospitalized eye trauma in Taiwan and investigate the role between principal and secondary diagnoses of such trauma.

**Methods:**

Nationwide fixed cohort study of 1,000,000 beneficiaries from the Taiwan Longitudinal Health Database was used and 4819 patients who were hospitalized for eye trauma during 1996-2010 were analyzed.

**Results:**

During 1996-2010, the incidence rates of hospitalized eye trauma (per 100 000 person-years) were 35.0 (95% confidence interval (CI), 34.0 to 36.0) for total diagnosis, 9.8 (95% CI, 9.3 to 10.3) for a principal diagnosis, and 25.3 (95% CI, 24.4 to 26.1) for a secondary diagnosis. The sex risk ratio was 3.1 for a principal diagnosis and 2.1 for a secondary diagnosis. The main causes of eye trauma were traffic accident, work accident, assault (among males <60 years of age), and falls (among elderly men and women). The proportion admitted to an ophthalmic department among those with a principal diagnosis of eye trauma (64.8%) was significantly higher than among those with a secondary diagnosis (2.3%) (p<.0001). Patients with a principal diagnosis of eye trauma had shorter hospital stays (7.1±10.2 days) and lower fatality (0.07%) than those with a secondary diagnosis of eye trauma (10.0±31.6 days and 0.3%, respectively).

**Conclusion:**

Data only from ophthalmic admissions tends to underestimate the true incidence rate of hospitalized eye trauma. Patients with a principal diagnosis of eye trauma had less severe injuries than did those with a secondary diagnosis.

## Introduction

Eye trauma is a common preventable cause of blindness. An estimated 55 million eye injuries occur annually worldwide, of which 19 million result in permanent unilateral vision loss and 1.6 million in blindness [1]. Due to the potential to threaten eyesight, severe injuries resulting in hospitalization are of most concern.

From an epidemiologic perspective, previous studies of eye trauma had several drawbacks. First, the role of principal and secondary diagnoses has not been adequately investigated, as some studies did not collect comprehensive diagnostic data [2–6]. Second, underestimation of the incidence rate of eye trauma is likely when patients are recruited only from ophthalmic departments [2–7].

We estimated the rate of hospitalized eye trauma in Taiwan and compared the characteristics of eye trauma (including sex and age distribution, E-code, admitting department, duration of hospital stay, fatality, and follow-up rate in ophthalmic departments) between principal and secondary diagnosis.

## Materials and Methods

### Data source

The primary data source of this study was the Taiwan Longitudinal Health Insurance Database (LHID). Taiwan has a population of approximately 23 million. In 1995, Taiwan established a compulsory single-payer National Health Insurance system and reached 99% coverage of the population by 2012 [8]. For research purposes, 1 000 000 beneficiaries were randomly sampled from the entire beneficiaries in 1996–2000, and their registration and claims data during 1996–2010 are available in LHID. There are no significant sex differences between the LHID and entire research dataset [9]. Identification numbers were de-identified before they were released for research to ensure confidentiality.

This study was approved by the Institutional Review Board at Chang Gung Memorial Hospital, Taiwan (101-4675B). Because all data were completely anonymous, informed patient consent was not required.

### Study design

This was population-based fixed cohort study. Records were included if they showed a diagnosis of eye trauma (International Classification of Diseases, Ninth Revision, Classification Modification, [ICD-9-CM]: 802.6–802.7, 870.0–870.9, 871.0–871.9, 918.0–918.9, 921.0–921.9, 930.0–930.9, 940.0–940.9, 950.0–950.9, 951.0–951.1, and 951.3) and hospitalization during 1996–2010. A principal diagnosis of eye trauma was defined as any record with the first diagnosis corresponded to 1 of the above ICD-9-CM codes. A secondary diagnosis of eye trauma was defined as any record in which any 1 of the 4 secondary diagnoses included 1 of the above ICD-9-CM codes.

### Statistical analysis

Incidence rate was calculated as the numbers of new cases from 1996 through 2010 divided by person-years involved. The 95% confidence intervals (CIs) of incidence rate were provided, assuming a Poisson distribution. To compare our incidence rate with those from other studies, direct age-standardization was done, using the new World Health Organization Standard Population [10]. The sex risk-ratio (RR) was defined as the incidence rate for males divided by the incidence rate for females. The independent t-test or χ^2^ test was used to compare data between two diagnoses, when appropriate. The significance level was defined as 0.05.

## Results

During 1996–2010, 4819 patients had hospitalized eye trauma, corresponding to an incidence rate of total diagnosis of eye trauma of 35.0 per 100 000 person-years (95% CI, 34.0–36.0). Among these 4819 patients, 27.9% received a principal diagnosis and 72.1% received a secondary diagnosis. Thus, the rate of eye trauma (per 100 000 person-years) was 9.8 (95% CI, 9.3–10.3) for a principal diagnosis and 25.3 (95% CI, 24.4–26.1) for a secondary diagnosis. The ratio of secondary to principal diagnoses of eye trauma was 2.58 (95% CI, 2.51–2.66).

The incidence rate (per 100 000 person-years) of a principal diagnosis was 14.7 (95% CI, 13.8–15.6) among males and 4.7 (95% CI 4.2–5.3) among females, which corresponds to a sex RR of 3.1 (95% CI 2.7–3.5). The incidence rate (per 100 000 person-years) of a secondary diagnosis was 33.8 (95% CI, 32.5–35.2) among males and 16.4 (95% CI 15.4–17.3) among females, which corresponds to a sex RR of 2.1 (95% CI 1.9–2.2).

**Fig 1:**
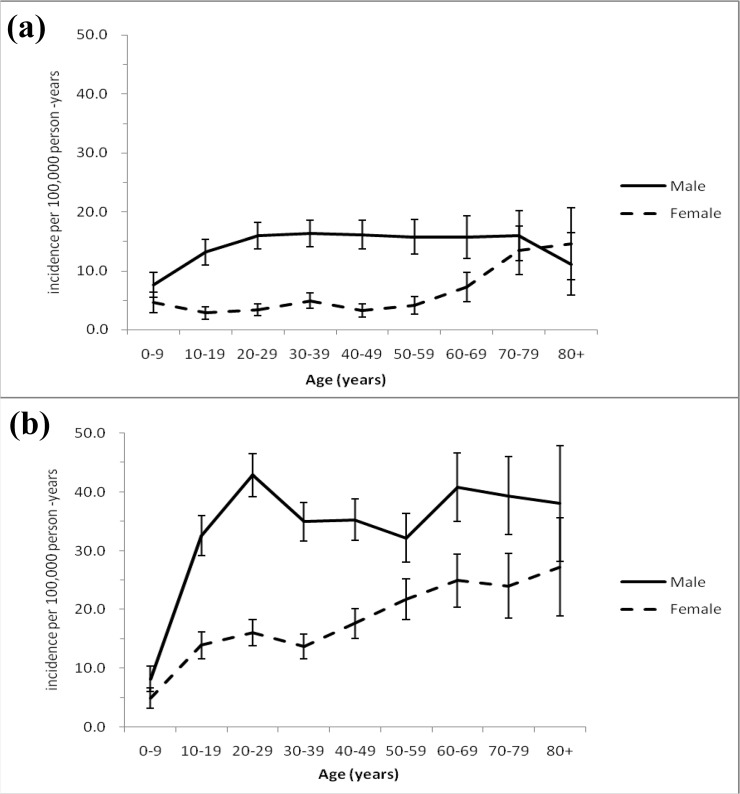
Age-specific incidence rate (per 100 000 person-years) of hospitalized eye trauma as a principal diagnosis (a) and secondary diagnosis (b) by sex, Taiwan (1996–2010), and error bar indicated 95% confidence interval.

Age-specific incidence rates of two diagnoses substantially differed between males and females (Fig 1A and 1B). For principal diagnoses, the rate among males rose from age 0–29 years, plateaued until age 70–79 years, and dropped after age 80 years. Among females, the rate gradually rose and reached a local peak at age 30–39 years and another peak at age 70–79 years. The highest sex RRs were 5.0 (95% CI 3.5–7.1) at age 40–49 years, and the lowest sex RR were 0.8 (95% CI 0.4–1.4) at age ≥80 years. For secondary diagnoses, both males and females had peaks (per 100 000 person-years) at age 20–29 years and 60–69 years. The highest sex RRs were 2.7 (95% CI, 2.3–3.2) at age 20–29, and the lowest sex RR were 1.4 (95% CI, 0.9–2.1) at age ≥80 years.

For eye-related trauma, the most common principal diagnosis was "open wound of eyeball" (41.6%), followed by "open wound of ocular adnexa" (23.0%) and “contusion of eye and adnexa" (15.3%) (Table 1). The most common secondary diagnosis was "contusion of eye and adnexa" (48.7%), followed by "open wound of ocular adnexa" (29.2%) and "orbital floor fractures" (7.5%).

**Table 1:** Frequency of diagnoses of hospitalized eye trauma, Taiwan (1996–2010).

Diagnosis (ICD-9-CM^1^)	Principal diagnosis		Secondary diagnosis	
**Eye-related trauma**				
Open wound of eyeball (871.0–871.9)	560	(41.6%)	139	(4.0%)
Open wound of ocular adnexa (870.0–870.9)	309	(23.0%)	1,014	(29.2%)
Contusion of eye and adnexa (921.0–921.9)	206	(15.3%)	1,693	(48.7%)
Orbital floor fractures (blow out) (802.6–802.7)	129	(9.6%)	259	(7.5%)
Burn confined to eye and adnexa (940.0–940.9)	75	(5.6%)	48	(1.4%)
Injury to optic nerve and pathways (950.0–950.9)	29	(2.2%)	196	(5.6%)
Superficial injury of eye and adnexa (918.0–918.9)	24	(1.8%)	179	(5.2%)
Foreign body on external eye (930.0–930.9)	10	(0.7%)	96	(2.8%)
Injury to oculomotor, trochlear and abducens nerve (951.0, 951.1, 951.3)	3	(0.2%)	65	(1.9%)
**Non-eye-related trauma**				
Fracture of facial bones (802)	179	(13.3%)	746	(21.5%)
Open wound, other than head and eye (872–899)	174	(12.9%)	1,335	(38.4%)
Head injury (800, 801, 803, 804, 850–854)	143	(10.6%)	2,052	(59.1%)
Fracture of spine, trunk or limbs (805–829)	47	(3.5%)	751	(21.6%)

^1^ International Classification of Diseases, Ninth Revision, Classification Modification.

Non–eye-related trauma in patients with a principal diagnosis included “fracture of facial bones" (13.3%), “open wound other than head and eye" (12.9%), and “head injury" (10.6%) (Table 1). The most common secondary diagnoses were “head injury" (59.1%), followed by “open wound other than head and eye" (38.4%), “fracture of spine, trunk, or limbs" (21.6%), and “fracture of facial bones" (21.5%).

External causes of trauma, or E-codes, were available for 889 (66.1%) patients with a principal diagnosis of eye trauma and for 2416 (69.5%) patients with a secondary diagnosis of eye trauma (Table 2). Traffic accident was the most common cause for a principal diagnosis (20.4%) and a secondary diagnosis (47.2%). Of the traffic accidents, “motorcycles” was the predominant cause for both diagnoses. For principal diagnoses, the second most common cause was “falling objects" (10.0%), followed by “cutting and piercing" (7.7%) and “foreign body” (7.4%). For secondary diagnoses, the second most common cause was “falls" (9.3%), followed by “homicide" (6.8%).

**Table 2:** External causes of hospitalized eye trauma, Taiwan (1996–2010).

	Principal diagnosis	Secondary diagnosis	P
Number of subjects	1,345	3,474	
Traffic accident	274 (20.4%)	1,638 (47.2%)	<.0001
Motorcycles	128 (9.5%)	909 (26.2%)	
Falls	84 (6.3%)	323 (9.3%)	
Falling objects	135 (10.0%)	69 (2.0%)	
Cutting and piercing	104 (7.7%)	15 (0.4%)	
Foreign body	99 (7.4%)	14 (0.4%)	
Homicide	71 (5.3%)	235 (6.8%)	
Other (including sports-related injury)	122 (9.1%)	133 (3.5%)	
Missing	456 (33.9%)	1,058 (30.5%)	

**Fig 2:**
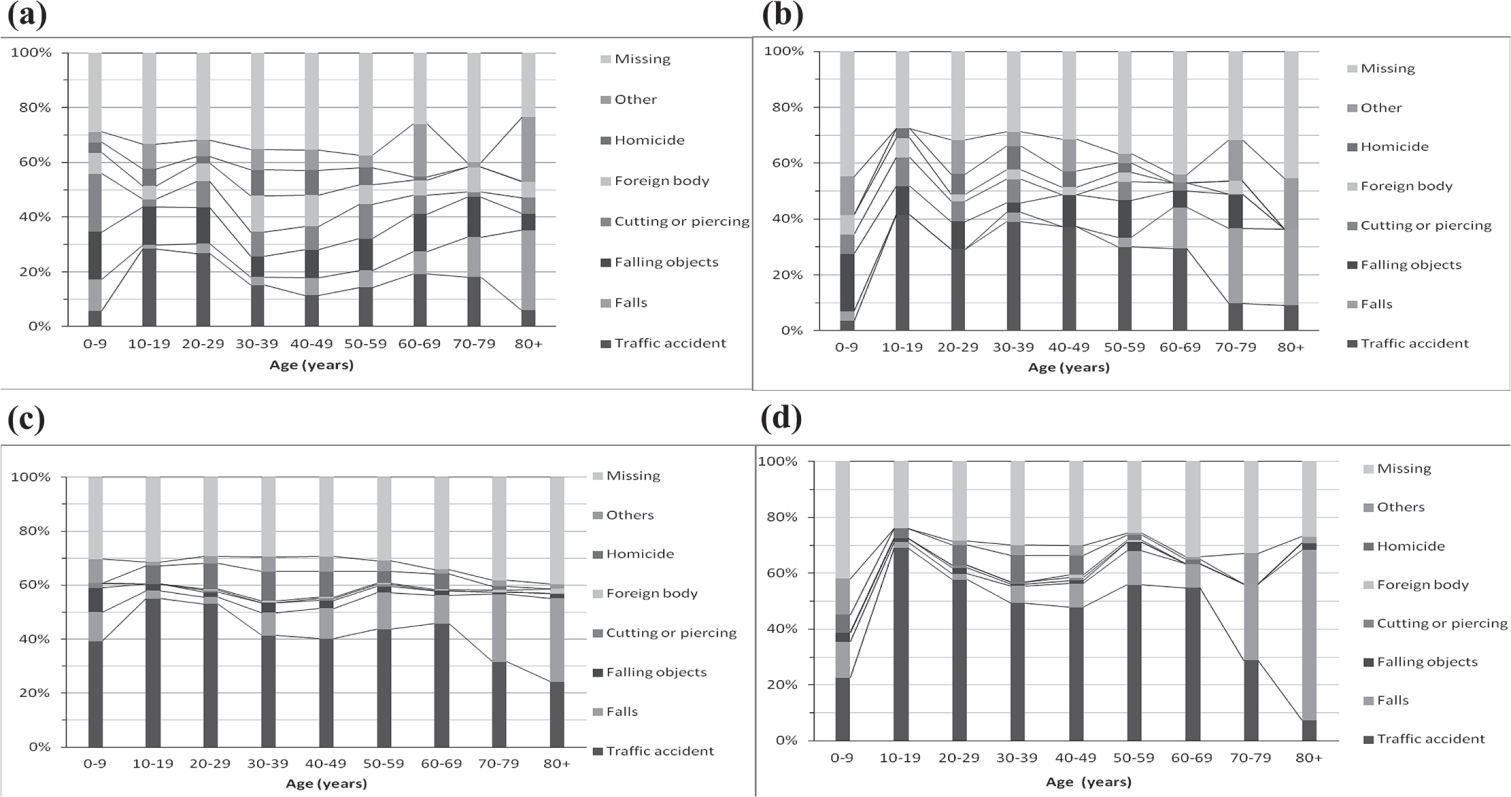
Cause-specific proportion of hospitalized eye trauma as a principle diagnosis among males (a) and females (b), and a secondary diagnosis among males (c) and females (d), Taiwan (1996–2010).

The age-specific distribution of E-codes (Fig 2A–2D) showed a peak for “traffic accident” at age 10–19 years, and low points at both ends of the age spectrum. In contrast, most falls occurred at the ends of the age spectrum, and the peak was seen for the oldest group. Obviously, these 2 causes combined had a predominant role in the secondary diagnosis of hospitalized eye trauma at all ages.

About 64.8% of patients who received a principal diagnosis of eye trauma were admitted to an ophthalmic ward, while only 2.3% who received a secondary diagnosis were admitted to an ophthalmic ward (p<.0001) (Table 3). The average duration of hospitalization among patients with a principal diagnosis (7.1±10.2 days) was significantly shorter than that for patients with a secondary diagnosis (10.0±31.6 days) (p<.0001). The longest mean duration of hospitalization was for patients with a secondary diagnosis who were admitted to a non-ophthalmic department (10.1±31.9 days). There was no significant difference in fatality between primary diagnosis (0.07%) and secondary diagnosis (0.3%). The number of outpatient visits to an ophthalmic clinic within 6 months after discharge was higher for a principal diagnosis (5.3±5.5) than for a secondary diagnosis (1.2±2.5) (p<.0001), mainly due to the high number of such visits among those who were admitted to ophthalmic departments.

**Table 3:** Hospitalization and discharge characteristics for hospitalized eye trauma, Taiwan (1996–2010).

	Principal diagnosis				Secondary diagnosis				
	Total	Ophthalmic	Non-ophthalmic	P^1^	Total	Ophthalmic	Non-ophthalmic	P^1^	P^2^/P^3^
Number of subjects	1,345	871 (64.8%)	474 (35.2%)		3,474	79 (2.3%)	3,395 (97.7%)		
Duration of stay (d)				.4613				<.0001	.2356/.0042
median	6.0	6.0	5.0		6.0	5.0	6.0		
mean±SD	7.1±10.2	6.9±5.1	7.5±15.7		10.0±31.6	8.3±10.3	10.1±31.9		
(95% CI)	(6.6–7.6)	(6.6–7.2)	(6.1–8.9)		(8.9–11.1)	(6.0–10.6)	(9.0–11.2)		
Fatality	1 (0.07%)	0 (0.0%)	1 (0.2%)	.3524	12(0.3%)	0 (0.0%)	12 (0.4%)	1.0000	— /1.0000
(95% CI)	(0.02–0.4%)	—	(0.06–1.2%)		(0.09–0.6%)	—	(0.1–0.6%)		
Outpatient visit to ophthalmic clinic within 6 months after discharge				<.0001				<.0001	.0057/.3955
mean±SD	5.3±5.5	7.5±5.4	1.2±2.4		1.2±2.5	6.1±4.1	1.1±2.4		
(95% CI)	(5.0–5.6)	(7.1–7.9)	(1.0–1.4)		(1.1–1.3)	(5.2–7.0)	(1.0–1.2)		

^1^ Comparison between ophthalmic and non-ophthalmic departments.

^2^ Comparison between principal and secondary diagnoses among patients admitted to ophthalmic department.

^3^ Comparison between principal and secondary diagnosis among patients admitted to non-ophthalmic department.

## Discussion

### Comparing incidence rate

The incidence rate of total diagnosis of hospitalized eye trauma (per 100 000 person-years) was higher in this study (35.0) than in studies that recruited patients from both ophthalmic and non-ophthalmic departments (e.g. 27.3 in Maryland [11] and 29.1 in the US [12]) (Table 4). We calculated age-standardized incidence rates (per 100 000 person-years), and the resulting values were 32.9 for this study, 27.6 for Maryland, and 27.5 for the US. The present rate remains highest, perhaps because of the high rate of traffic accidents in Taiwan. For instance, the proportion of total diagnosis associated with traffic accidents in this study (39.7% = (274+1638)/4819; Table 2) was higher than that in the US (30.9%) [12].

**Table 4:** Summary of studies about hospitalized eye trauma by published year.

Authors	Country	Study design / Study years / Number of patients	Hospital eye trauma rate / standardized rate (per 100,000 person-year)			2^nd^: 1^st^	RR = (R_M_/R_F_)	M:F	Cause
			1^st^	2^nd^	Total				
**Tielsch, Parver et al. 1989**	MD, USA	Population-based/ 1979–1986 / 1st: 4535; total: 9373	13.2/ 13.3	—	27.3/ 27.6	48.4%: 51.5% = 1.06	—	—	1st: assault, traffic accident; T: traffic accident, falls
**Klopfer, Tielsch et al. 1992**	USA	Nation-based (cover 84% of hospitals) / 1984–1987 / 1st: 31766; total: 69729	13.2/ 13.3	—	29.1/ 27.5	45.4%: 54.6% = 1.20	3.2(1st), 2.3(T)	—	1st: guns, traffic accident, foreign body; T: traffic accident, homicide, falls
**Desai, MacEwen et al. 1996**	Scotland	Nation-based (18 Oph- hospitals) / 1991 / 415	8.14	—	8.14/ 8.5	—	5.41	—	Assault, sport, traffic accident
**Baker, Wilson et al. 1999**	CA, USA	Population-based (workers only) / 1988 / 1st: 269; 2nd: 186	—	—	1.76/ 2.88	59.1%: 41.9% = 0.71	—	—	1st: foreign body, cutting, erosive; 2nd: traffic accident, assault, falls
**Wong and Tielsch 1999**	Singapore	Nation-based / 1991–1996 / 2029	12.6/ 12.1	—	—	—	3.6 (Chinese) 4.2 (Malays) 7.9 (Indians)	—	—
**Mela, Mantzouranis et al. 2005**	Greek	Two oph-hospitals / 1993–2002 / 937	—	—	—	—	—	4.05	Work, home, traffic accident
**Chang, Chen et al. 2008**	Kaohsiung, Taiwan	One oph-hospital / 2001–2002 / 160	—	—	—	—	—	4.93 (work) 3.1(nonwork)	Work, contusion
**Cillino, Casuccio et al. 2008**	W Sicily, Italy	One oph-hospital / 2001–2005 / 298	4.9	—	—	-	—	5.5	—
**Saeed, Khan et al. 2010**	South-east Ireland	One oph-hospital / 2001–2007 / 517	—	—	19.4, 22.0 (20002), 19.0 (2006)	—	—	6.6	Work, home
**Cao, Li et al. 2012**	Chaoshan, China	Three oph-hospitals / 2001–2010 / 3664 injured eyes; 3559 patients	—	—	27.7	—	—	5.2	Open-globe, work
**This study**	Taiwan	Nation-based / 1996–2010 / 1st: 1345; 2nd: 3474	9.8/ 9.3	25.3/ 23.5	35/ 32.9	—	3.1 (1st), 2.1 (2nd)	—	—

1^st^ represents principal diagnosis,

2^nd^ represents secondary diagnosis,

T represents total diagnosis and RR for risk ratio.

The incidence rate of a principal diagnosis of hospitalized eye trauma in this study (9.8 per 100 000 person-years) was lower than in studies that recruited patients from both ophthalmic and non-ophthalmic departments (US: 13.2 [11, 12]; and Singapore: 12.6 [13]). After direct standardization, the incidence in this study (9.3) was still lower than rates in the US (13.3) and Singapore (12.1). The lower incidence in Taiwan was probably due to the fact that some patients with multiple injuries and life-threatening trauma were prioritized and admitted to non-ophthalmic departments. In this study, only 64.8% of patients with a principal diagnosis of eye trauma were admitted to an ophthalmic department (Table 3).

The ratio of secondary to principal diagnoses of eye trauma was 2.6, approximately 1-fold that reported in 2 US studies [11, 12]. One possibility is that many hospitals in Taiwan during the past 2 decades have included trauma specialists in their emergency department; thus, patients with eye trauma and multiple injuries, and patients without open wounds of the eyeball or ocular adnexa, were mostly admitted to non-ophthalmic departments. In this study, 64.8% of patients with a principal diagnosis of eye trauma were admitted to an ophthalmic department, but only 2.3% of patients with a secondary diagnosis of eye trauma were admitted to an ophthalmic department (Table 3).

Among studies that included patients only admitted to ophthalmic departments, incidence rates (both total and principal diagnoses) were consistently lower than those in this study. The incidence rate (per 100 000 person-years) for total diagnosis of eye trauma was 8.14 for Scotland [7], 19.4 for southeast Ireland [5], and 27.7 for the Chaoshan region of China [6]. The incidence rates (per 100 000 person-years) for a principal diagnosis of eye trauma was 4.9 for southern Italy [4]. The proportion of patients with hospitalized eye trauma admitted to ophthalmic departments in those studies was not reported. In this study, only 20% of patients (i.e. (871 + 79)/4819; Table 3) with eye trauma were admitted to an ophthalmic department. More importantly, data only from ophthalmic admissions tend to underestimate the true incidence rate of hospitalized eye trauma.

### Causes of trauma by sex and age

Sex and age are important demographic factors in hospitalized eye trauma. In general, males have a consistently higher risk than females of eye trauma. Previous studies reported male/female ratios ranging from 3.1 to 6.6 [2–6]. The present sex RR was 3.1 in the principal diagnosis group, similar to that in the US (3.2) [11] but lower than in Scotland (5.4) [7] and in Singapore (Chinese, 3.6; Malays, 4.2; Indians, 7.9) [13].

When age and sex are considered together, males do not always have a higher risk of eye trauma than females. Among elderly adults, men and women in this study had an equal risk of a principal diagnosis of eye trauma, similar to previous studies [11–13]. Falls were responsible for most eye trauma among elderly adults in US [11, 12] and this study (Fig 2A–2D).

Among younger people (age <60), most studies reported that incidence rates peaked at a younger age and decreased thereafter [11–13]. The peak age at secondary diagnosis was 20–29 years for both sexes in this study. Traffic accident is the predominate cause of a principal diagnosis of eye trauma among the US [11, 12] and this study (Fig 2A–2D). Furthermore, half of ocular trauma due to traffic accidents in this study was related to motorcycles, which explains the peak for traffic accidents at age 10–29 years.

In contrast, the trend in the incidence rate of a principal diagnosis of eye trauma among males in this study was unusual. It rose at age 20–29 years and later became stable, which differed from trends noted in previous studies [11–13]. The fact that other causes (falling objects, cutting or piercing, and foreign bodies; 7–10% each) were as important as traffic accidents explains this phenomenon (Fig 2A). We suspected that falling objects, cutting or piercing, and foreign bodies were work-related. The California study showed that the most common causes of work-related eye trauma were foreign-body or projectile objects (19%), transport vehicles (18%), and cutting or piercing objects (17%) [14]. The important role of work-related eye trauma among young and middle age men has been reported previously [3–5].

In addition to traffic accidents and work, 2 important causes of eye trauma among young people are assault [5, 11, 12] and sports [11]. Assault proportion was 18.4% in Maryland [11], 16.6% in the US (due to BB and air guns) [12], and 12.2% in southeast Ireland [5]. In this study, homicide was responsible for 6.9%, 9.1%, 6.7%, and 6.5% of principal and secondary diagnoses of eye trauma among men and women aged 20–59 years, respectively (Fig 2A–2D). For sports, the proportion of eye trauma among people aged 20–29 years was as high as that due to assault in Maryland [11] and due to traffic accident in southern Italy [4]. In southeast Ireland, sports is the third cause of eye trauma for people aged 16–64 years [5]. In this study, very few cases of eye trauma were sports-related (1.1% of principal diagnoses and 0.2% of secondary diagnoses; data not shown). Underreporting was very likely because of lack of financial incentives for hospitals, inadequate space on records, and insufficient details in the medical record of a hospital administrative database.

It is possible that the limited availability of E-codes constrained our analysis of the causes of trauma. However, E-codes were available in about two-thirds of the records, which is higher than 3 US studies (Maryland: 52.3% of principal diagnoses and 39.2% of secondary diagnoses [11]; US: 24% of principal diagnoses and 26% of all diagnoses [12]; California: 30.9% of principal diagnoses and 36.6% of secondary diagnoses [14]), but lower than studies of the Chaoshan region of China (89.4%) [6] and in southeast Ireland (98.6%) [5]. In our study, there was no difference in age or sex distribution between records with and without E-codes (data not shown). We thus assume that the incompleteness of E-codes does not alter our findings on the external causes of eye trauma [15].

### Type of trauma

The type of trauma (either eye- or non–eye-related) is related to the admitting department (ophthalmic vs. non-ophthalmic). We found a significant difference in type of trauma (eye- vs. non–eye-related) between principal and secondary diagnoses. Among principal diagnoses, the two most common eye-related types of trauma were open wound of the eyeball and open wound of the adnexa, similar to previous studies [4, 11–13]. It is unsurprising to see high proportions of such injuries as principal diagnoses, because open wounds of the eyeball are usually treated by ophthalmologists.

Among cases with non–eye-related trauma, we found that fracture of the facial bones, and open wounds of areas other than the head and eyes were equally common as principal diagnoses, while head injury and open wounds of areas other than the head and eyes were predominant among secondary diagnoses. Similar findings were noted in California study [14]. Among secondary diagnoses, the combination of head injury (59.1%) and open wounds of areas other than the head and eye (38.4%) was more frequent than contusion of the eye and adnexa (48.7%), which indicates that patients had more-severe trauma in areas other than the eye and were thus admitted to non-ophthalmic departments.

### Impact (hospital stay, fatality, and follow-up visits)

In this study, it was obvious that patients with a principal diagnosis of eye trauma were less severely injured than those with a secondary diagnosis, as the former had shorter hospital stays and a lower fatality rate. The longest hospital stays were seen among patients with secondary diagnoses who were admitted to non-ophthalmic departments. These patients usually had multiple injuries, which explains their longer stays. In this study, the duration of stay associated with a principal diagnosis (mean: 7.1±10.2 days; median: 6 days) was similar to that in a study of southern Taiwan [3] but longer than that in a Greek study (5.6±4.6 days) [2] and a study of southeast Ireland (5.0±19.7 days) [5]. When we limited our analysis to those admitted to an ophthalmologic department, the duration of stay for a principal diagnosis (6.9±5.1 days) was still longer, probably because neither patients nor physicians had an incentive to shorten length of stay, due to the coverage of the National Health Insurance Program in Taiwan [16, 17].

In this study, the fatality rate was 0.07% and 0.3% in patients with principal and secondary diagnoses, respectively, lower than that (0.4% and 0.7%) reported for a population of US workers [14]. However, patients with a principal diagnosis of eye trauma required more follow-up visits to ophthalmic clinics within 6 months after discharge than did those with a secondary diagnosis. After stratifying the data by admitting department, the number of follow-up visits to ophthalmic clinics was similar for patients with principal and secondary diagnoses, implying that eye trauma was of comparable visual outcome for these two groups.

### Study strengths and limitations

The strengths of this study include (1) a nationwide cohort which minimized selection bias, (2) the longitudinal design which permitted calculation of incidence, and (3) the inclusion of 1 principal diagnosis and (4) secondary diagnoses, which allowed stratification by principal diagnosis and secondary diagnosis of eye trauma.

The limitations of this study include (1) the inability to calculate the overall rate of ocular trauma (hospitalized and non-hospitalized), (2) lack of severity scores for eye trauma, (3) lack of detailed clinical and visual information to assess vision loss, (4) inappropriateness to compute time trend of the incidence rate from 1996 to 2010 because numbers of participants decline over time and aging problem over time for a fixed cohort [18].

## Supporting Information

S1 Checklist Strobe Checklist. (DOCX)

## References

[1] Negrel AD, Thylefors B. The global impact of eye injuries. Ophthalmic Epidemiol. 1998;5(3), 143–169. 9805347 10.1076/opep.5.3.143.8364

[2] Mela EK, Mantzouranis GA, Giakoumis AP, Blatsios G, Andrikopoulos GK, Gartaganis SP. Ocular trauma in a Greek population: review of 899 cases resulting in hospitalization. Ophthalmic Epidemiol. 2005;12(3), 185–190. 16036477 10.1080/09286580590964801

[3] Chang CH, Chen CL, Ho CK, Lai YH, Hu RC, Yen YL. Hospitalized eye injury in a large industrial city of South-Eastern Asia. Graefes Arch Clin Exp Ophthalmol. 2008;246(2), 223–228. 10.1007/s00417-007-0733-z 18180943

[4] Cillino S, Casuccio A, Di Pace F, Pillitteri F, Cillino G. A five-year retrospective study of the epidemiological characteristics and visual outcomes of patients hospitalized for ocular trauma in a Mediterranean area. BMC Ophthalmol. 2008;8. 10.1186/1471-2415-8-6 PMC2387139 18430231

[5] Saeed A, Khan I, Dunne O, Stack J, Beatty S. Ocular injury requiring hospitalisation in the South East of Ireland: 2001–2007. Injury. 2010;41(1), 86–91. 10.1016/j.injury.2009.01.118 19493528

[6] Cao H, Li L, Zhang M. Epidemiology of patients hospitalized for ocular trauma in the Chaoshan Region of China, 2001–2010. PLOS ONE. 2012;7(10), 1–7. 10.1371/journal.pone.0048377 PMC3485239 23118997

[7] Desai P, MacEwen CJ, Baines P, Minassian DC. Incidence of cases of ocular trauma admitted to hospital and incidence of blinding outcome. Brit J Ophthalmol. 1996;80(7), 592–596. 8795369 10.1136/bjo.80.7.592 PMC505551

[8] National Health Research Institutes, Taiwan. (Dec. 6, 2012). Universal Health Coverage in Taiwan updated on 2013-04-29. Available: http://www.nhi.gov.tw/Resource/webdata/20517_2_201304_National%20Health%20Insurance%201.pdf. Accessed 05 May 2013.

[9] National Health Research Institutes, Taiwan. Introduction to the National Health Insurance Research Database (NHIRD), Taiwan. Available: http://nhird.nhri.org.tw/date_01.html. Accessed 05 May 2013.

[10] Ahmad OB, Boschi-Pinto C, Lopez AD, Murry CJ, Lanzano R, Inoue M. (2001). Age standardization of rates: A new WHO standard Geneva: World Health Organization Available: http://www.who.int/healthinfo/paper31.pdf.

[11] Tielsch JM, Parver L, Shankar B. Time trends in the incidence of hospitalized ocular trauma. Arch. Ophthalmol. 1989;107(4), 519–523. 2705918 10.1001/archopht.1989.01070010533025

[12] Klopfer J, Tielsch JM, Vitale S, See LC, Canner JK. Ocular trauma in the United States. Eye injuries resulting in hospitalization, 1984 through 1987. Arch. Ophthalmol. 1992;110(6), 838–842. 1596232 10.1001/archopht.1992.01080180110037

[13] Wong TY, Tielsch JM. A population-based study on the incidence of severe ocular trauma in Singapore. Am J Ophthalmol. 1999;128(3), 345–351. 10511030 10.1016/s0002-9394(99)00167-1

[14] Baker RS, Wilson MR, Flowers CW Jr, Lee DA, Wheeler NC. A population-based survey of hospitalized work-related ocular injury: Diagnoses, cause of injury, resource utilization, and hospitalization outcome. Ophthalmic Epidemiol. 1999;6(3), 159–169. 10487971 10.1076/opep.6.3.159.1505

[15] Sniezek JE, Finklea JF, Graitcer PL. Injury coding and hospital discharge data. JAMA. 1989;262(16), 2270–2272. 2795809

[16] Huang SK, Tsai SL, Hsu M. T. Ensuring the sustainability of the Taiwan National Health Insurance. J Formos Med Assoc. 2014;113: 1–2. 10.1016/j.jfma.2013.08.010 24060196

[17] Lai MS. National health insurance and the way leading to better diabetes care in Taiwan. Is there a role of comprehensive analyses of the claims data? J Formos Med Assoc. 2012;111:587–588. 10.1016/j.jfma.2012.08.016 23217593

[18] Rothman KJ. Modern epidemiology Boston/Toronto: Little, Brown and Company; 1986.

